# Using geographically weighted Poisson regression to examine the association between socioeconomic factors and hysterectomy incidence in Wallonia, Belgium

**DOI:** 10.1186/s12905-021-01514-y

**Published:** 2021-10-26

**Authors:** Aline Poliart, Fati Kirakoya-Samadoulougou, Mady Ouédraogo, Philippe Collart, Dominique Dubourg, Sékou Samadoulougou

**Affiliations:** 1 Centre de Recherche en Epidémiologie, Biostatistiques et Recherche Clinique, Ecole de Santé Publique, Université Libre de Bruxelles, 1070 Brussels, Belgium; 2 Agence pour une Vie de Qualité (AVIQ), 6061 Charleroi, Belgium; 3 Evaluation Platform on Obesity Prevention, Quebec Heart and Lung Institute, Quebec, QC G1V 4G5 Canada; 4 Centre for Research on Planning and Development, Université Laval, Quebec, QC G1V 0A6 Canada

**Keywords:** Geographically weighted Poisson regression, Wallonia, Hysterectomy, Socioeconomic factors

## Abstract

**Background:**

Various studies have investigated geographical variations in the incidence of hysterectomy in Western countries and analyzed socioeconomic factors to explain those variations. However, few studies have used spatial analysis to characterize them. Geographically weighted Poisson regression (GWPR) explores the spatially varying impacts of covariates across a study area and focuses attention on local variations. Given the potential of GWPR to guide decision-making, this study aimed to describe the geographical distribution of hysterectomy incidence for benign indications in women older than 15 years old (15+) at the municipal level in Wallonia (southern region of Belgium) and to analyze potential associations with socioeconomic factors (‘Education/training’, ‘Income and purchasing power’ and ‘Health and care’) influencing the use of this surgery.

**Methods:**

We carried out an ecological study on data for women aged 15+ living in one of the 262 Walloon municipalities who underwent hysterectomies for benign indications between 2012 and 2014. We linked standardized hysterectomy rates to three municipal-level socioeconomic factors (‘Education/training’, ‘Income and purchasing power’ and ‘Health and care’). Then, a Poisson regression model and a GWPR were applied to study the relationships between hysterectomy incidence and socioeconomic covariates in Wallonia.

**Results:**

The hysterectomy rate varied across the region. The Poisson regression revealed a positive and significant association between the hysterectomy rate and ‘Income and purchasing power’, and a negative and significant association between hysterectomies and ‘Health and care’. The same associations were seen in the GWPR model. The latter demonstrated that the association between hysterectomies and ‘Education and training’ ranged from negative to positive over the study area.

**Conclusions:**

Hysterectomy incidence was shown to have nonstationary relationships with socioeconomic factors. These results support the development of targeted interventions for a more appropriate use of this surgery.

## Background

It has been estimated that around 20% of women in Western countries will undergo hysterectomies by the age of 60 [1–3]. A hysterectomy is usually performed for benign indications, such as endometriosis, menorrhagia, metrorrhagia, leiomyomas, genital prolapse and chronic pain [4, 5]. Hysterectomies for benign conditions are a common and effective form of elective surgery in Western countries and usually performed well. However, they have the potential to affect women’s physical, emotional, sexual and social wellbeing [4, 6]. In addition, as for all surgeries, short- and long-term complications can occur, causing subsequent healthcare costs [5, 7]. The surgical route influences postoperative wellbeing and complications. Minimally invasive approaches, laparoscopy and the vaginal route, are known to decrease the length of hospitalization and shorten postoperative recovery versus the abdominal route [7, 8]. Moreover, outpatient hysterectomy surgery is infrequent (0.64%), and the costs increase with the length of hospital stay [7, 9]. Various alternatives to radical surgery exist: less invasive laparoscopic (laparoscopic myomectomy) and hysteroscopic (transcervical myoma resection, transcervical endometrium resection) treatments are available, but also endoscopic treatments, MRI guided focused ultrasound treatments, hormonal drug therapies, levonorgestrel-releasing intrauterine devices and uterine artery embolizations. A hysterectomy is recommended when other treatment options have failed, are contraindicated, or are declined by the woman [4, 10, 11]. Since the 1970s, numerous studies have shown variation in hysterectomy practices between countries or regions within countries [7, 12, 13]. Those regional variations are potentially a sign of the nonoptimal use of resources or healthcare inequalities [8, 14]. It is, therefore, important to identify the causes of the unexplained overuse of hysterectomies in some regions.

Socioeconomic and environmental factors play an important role in the regional variations of surgery use. The socioeconomic factors commonly associated with variations in hysterectomy rates include level of education, employment, income and social status, while the associated environmental factors include medical facility or healthcare provider characteristics, neighborhood, and living in an urban or rural area [15–19]. Hysterectomies seem to be higher among women with lower socioeconomic statuses or those living in the most deprived areas [17–22].

In Belgium, social security covers most of the cost of the surgery [8, 9]. For benign conditions, conservative treatments are available, and a hysterectomy should be the last resort when no other treatment option can help the patient. Two studies from Belgium have investigated the variation of hysterectomy rates and explanative factors: one prepared by the Belgian Christian Mutuality [1], from 1994 to 1997, and the other by the Belgian Health Care Knowledge Center (KCE), from 1997 to 2002 [9]. Both studies highlighted important regional differences, where Flanders had a higher incidence of hysterectomy than Wallonia and Brussels. The variables related to medical facilities, e.g., the density of general practitioners or gynecologists, only weakly explained the variations. However, the two reports found more interventions for patients in the lower socioeconomic categories in terms of education or social class [1, 9]. For this reason, better surveillance and community-driven approaches applied at the regional level remain essential for these surgical procedures.

Traditional regression methods, such as generalized linear models (GLMs), are generally used in health studies to assess elective surgery disparities and their associated risk factors, such as socioeconomic or environmental factors [2, 6, 10, 15–18, 20, 23–25]. However, the relationship between a health outcome and its explanative factors can differ within the same region of a country. Techniques such as the GLM do not consider this spatial variation and can lead to biased results and poor guidance for healthcare professionals and the population. In order to consider spatial variation, spatial modeling techniques have been developed, including geographically weighted Poisson regression (GWPR): a spatial modeling technique that incorporates the nonstationary spatial structures of data into statistical models [26, 27]. It enables the analysis and mapping of local spatial associations between a health outcome and socioeconomic variables, at a small-area level. Identifying municipalities with high hysterectomy incidence and their associated socioeconomic factors would help in developing locally targeted approaches to decrease the number of interventions in at-risk populations.

In Belgium, spatial analysis has been rarely used in public health research. Given the potential of this type of analysis to guide decision-making, this study, using GWPR, aimed to describe, with the most recent data, the geographical distribution of the hysterectomy incidence at the municipality level in Wallonia, Belgium, and to analyze potential associations with socioeconomic factors (‘Education/training’, ‘Income and purchasing power’ and ‘Health and care’) influencing the use of this surgery.

## Methods

### Study site and design

We conducted an ecological study on data encompassing the Wallonia region. Wallonia is one of the three regions of Belgium, located in the southern part of the country. It accounts for 55% of Belgium's territory but only one-third of its population. The estimated population was 3,576,325 inhabitants on 1st January 2014. Wallonia has a territory of 16,844 km^2^, administratively divided into 5 provinces, 20 districts and 262 municipalities. Each municipality is defined by a Numeric System Identifier (NSI) code, a string of 5 characters: the first character identifies the province; the second, the district; and the last three, the unique code of the municipality.

### Data source

Hysterectomy counts were extracted from the Minimum Hospital Summary (RHM) and obtained from the Walloon Agency for a Life of Quality (AViQ). The RHM is a compulsory registration of hospital activities in Belgium. We obtained data from women older than 15 years (15+) who underwent hysterectomies between January 2012 and December 2014. The RHM contains details on the diagnosis, procedure, age and place of residence for the patient. The procedure and diagnosis codes in the RHM follow the International Classification of Diseases (ICD); data from 2012 to 2014 were coded with version 9. The procedures were collapsed into two surgical route categories as follows: abdominal hysterectomies (ICD-9 683, 684 and 686, including Laparoscopic supracervical hysterectomy, Laparoscopic total abdominal hysterectomy and Laparoscopic radical abdominal hysterectomy) and vaginal hysterectomies (ICD-9 685 and 687, including Laparoscopically assisted vaginal hysterectomy and Laparoscopic radical vaginal hysterectomy). We set up three categories of diagnosis according to the ICD-9: uterine disorders including uterine leiomyomas and benign neoplasms of the female genital tract (ICD-9 2180–2219), endometriosis (ICD-9 6170–6179), and genital prolapse (ICD-9 6180–6189). The selection of diagnoses was limited to the codes for benign indications; life-threatening conditions (e.g., cancerous uterine tumors or bleeding after delivery) have fewer therapeutic options.

Population and demographic data were obtained from the national census of Belgium [28]. The population means the total number of women aged 15+ living in Belgium. We assumed that the Belgian population is stable and used 2014 as the reference year.

### Study variables

#### Response variable

The variable of interest was the number of hysterectomies in women aged 15+ in Wallonia. To take into consideration the number of women aged 15+ in each spatial unit, we calculated the standardized incidence ratio (SIR) for each of the 262 municipalities.

#### Independent variables

We extracted our data from online public data provided on the Walloon Institute of Evaluation, Prospective and Statistics (IWEPS) website [29]; all the independent variables were available at the municipal level for Wallonia. We selected three independent variables from the Index of Conditions of Well-Being (ICWB). Of note, the ICWB is a synthetic indicator that measures the material conditions and quality of life in the 262 Walloon municipalities using data available at the municipal level. The ICWB is an average of 60 standardized indicators grouped into 36 subdimensions, then into 19 dimensions, and, finally, into 8 families. The ICWB calculation and data broken down by municipality are available on the IWEPS website for the year 2014.

From the ICWB, we extracted three variables of the 19 dimensions scores. All the scores ranged between 0 and 1. The following indicators were selected from the ICWB dimensions, as they are self-explanatory with regard to the socioeconomic and environmental factors often described in the literature: ‘Health and care’, ‘Education/training’ and ‘Income and purchasing power’. The ‘Health and care’ indicator includes the following variables: ‘Life expectancy at birth Men & Women’, ‘Number of years of life lost Men and Women at age 70’, ‘Percentage of people declared chronically sick’, ‘Percentage of people recognized as medically disabled’, ‘Prevalence of diabetes’, ‘Offer of general practitioners, independent nurses and physios in the municipality’, ‘Pedestrian access to a pharmacy’, ‘Access to a hospital equipped with an emergency mobile resuscitation unit (specialist emergency care)’, and ‘Access to a medical centre’; ‘Education/training’: ‘Percentage of pupils “on time” in secondary education’, and ‘Access to a nursery or primary school’; and ‘Income and purchasing power’: ‘Median income per declaration’, ‘Percentage of recipients of social assistance in the population aged 18 and above’, ‘Percentage of defaulting borrowers’, and ‘Proportion of people in collective debt settlement’.

Table 1 presents the descriptive statistics of the three independent variables. The minimum value of the three covariates was 0, and the maximum was 1, with a mean greater than or equal to 0.50.

**Table 1:** Descriptive statistics of independent variables

Variables	Min	Max	Mean	SD
Health and care	0.00	1.00	0.59	0.18
Education/training	0.00	1.00	0.57	0.15
Income and purchasing power	0.00	1.00	0.50	0.18

*SD* standard deviation

**Fig. 1:**
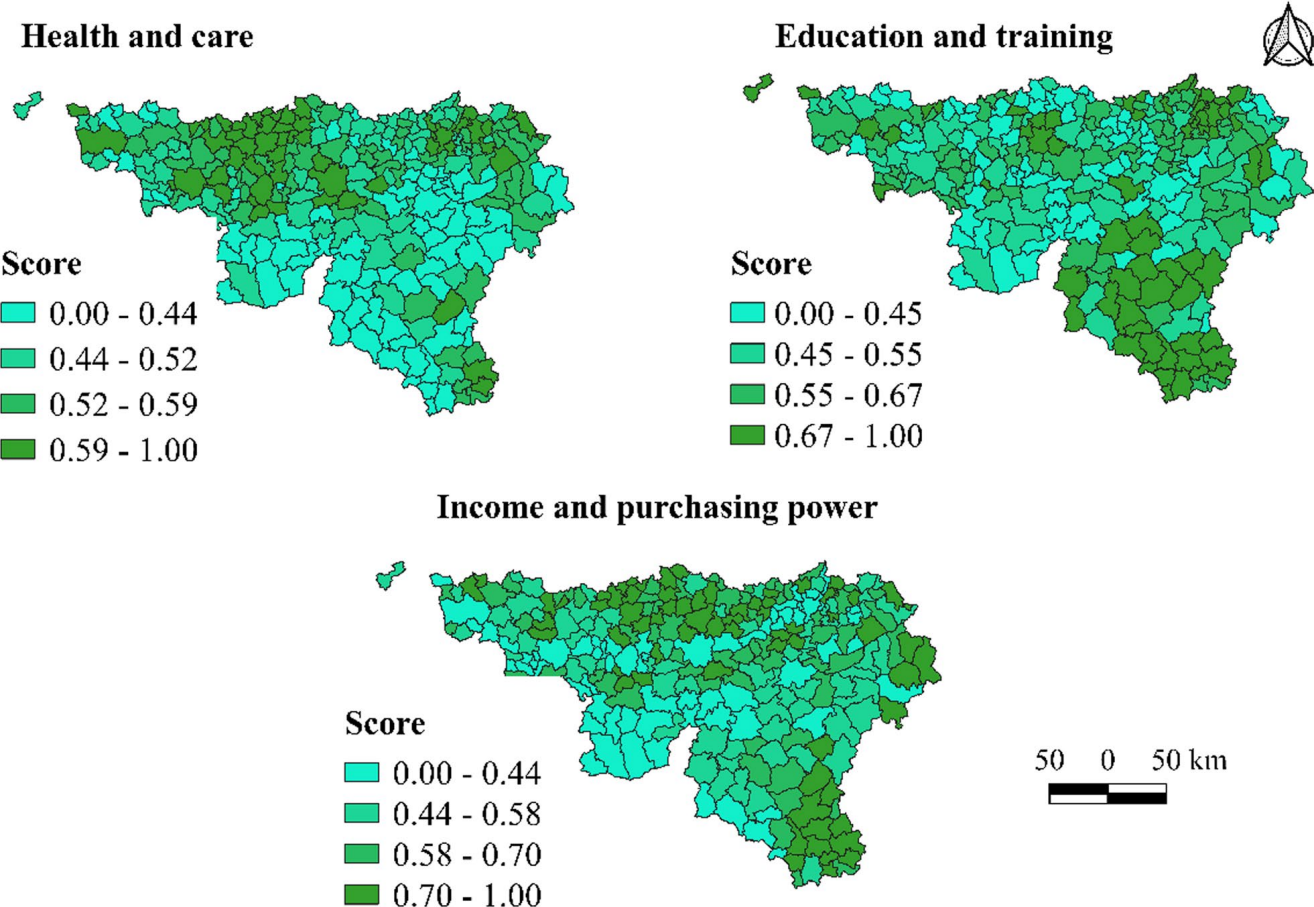
Spatial distribution of the index of conditions of well-being (ICWB) dimensions scores by municipalities ( Source: authors)

Figure 1 presents the spatial distribution of the three independent variables’ (ICWB dimensions) scores by municipality. For the ‘Health and care’ dimension, 67 municipalities had scores lower than 0.5 out of 1, and 11 had scores higher than 0.8. For the ‘Education/training’ dimension, 125 municipalities had scores lower than 0.5 out of 1, and 12 had scores higher than 0.8. For the ‘Income and purchasing power’ dimension, 75 municipalities had scores lower than 0.5 out of 1, and 19 had scores higher than 0.8.

### Statistical analysis

Two approaches were used to analyze the data: (1) We used descriptive statistics to characterize the response and independent variables. We categorized the SIRs of the 262 municipalities into 3 groups according to their values, and we mapped the 3 groups. (2) We performed a GLM with a log link that is Poisson regression model and a geographically weighted Poisson regression (GWPR) analysis to explore the associations between hysterectomies and socioeconomic factors.

#### Estimation of the standardized incidence ratio (SIR)

We estimated the age-standardized crude rate of hysterectomy by women's ages. The incidence of reported hysterectomy cases was measured as the number of new hysterectomy cases reported in a given year per 100,000 women. The confidence interval (CI) of the reported incidence was obtained by assuming that the observed incident cases followed a Poisson distribution.

$y_{i}$ is the number of observed hysterectomy cases, and $E_{i}$ is the expected number of hysterectomy cases for municipality $i$, where $i = 1, 2, \ldots , 262$. The SIR by age and municipality $i$ was defined as follows:

1 $${\text{SIR}}_{{\text{i}}} = \frac{{{\text{y}}_{{\text{i}}} }}{{{\text{E}}_{{\text{i}}} }} = \uptheta_{{\text{i}}} ,$$

The calculation of $E_{i}$ was based on the overall incidence rate $r_{k}$ applied to the age structure of the population of women aged 15+ in Belgium. Thus, the expected number of cases was calculated using the standardized incidence rate $r_{k}$ per age group. This rate was normalized by multiplying it by the proportion derived from the reference population, which was the age distribution of the Belgian population of 2014. The age groups were 20–34, 35–44, 45–49 and 50+. The standardized rate was then multiplied by the female population $p_{ki}$ within this age group in municipality $i$, where $k$ is the age group. The SIRs were then mapped by municipality, such that:

2 $${\text{r}}_{{\text{k}}} = \frac{{\mathop \sum \nolimits_{{\text{k}}} {\text{y}}_{{\text{k}}} }}{{\mathop \sum \nolimits_{{\text{k}}} {\text{p}}_{{\text{k}}} }}$$

where $k$ is the age group.

3 $$E_{i} = \mathop \sum \limits_{{\text{k}}} {\text{r}}_{{\text{k}}} \uprho_{{{\text{ki}}}}$$

The SIR is a standardized indicator of the incidence rate, and it varies around 1; if it is greater than, equal to, or less than 1, the observed number of cases is higher than, exactly equal to, or lower than that expected, respectively [30].

#### Modeling

In this study, a Poisson regression and GWPR were used to model the association between hysterectomies and the selected socioeconomic factors.

##### Poisson regression model

First, we implemented a Poisson regression model considering spatial autocorrelation to estimate the overall association between the three variables (‘Education/training’, ‘Income and purchasing power’ and ‘Health and care’) and the hysterectomy rate in women aged 15+ in Wallonia. This implementation allowed a global assessment of the existence of relationships, as well as their directions, between the different covariates and the hysterectomy rate in women before analyzing the effect of the association by municipality.

The formulation of the model was as follows:

4 $$\ln \left( {\text{Y}} \right) = \ln \left( {E_{i} } \right) + \upbeta_{0} + \upbeta_{1} \left( {{\text{ET}}} \right) + \upbeta_{2} \left( {{\text{IPP}}} \right) + \upbeta_{3} \left( {{\text{HC}}} \right) + \upvarepsilon$$

where *E*_*i*_ is the expected number of hysterectomy cases by municipality (i), β_0_ is the intercept, β (β_1_, β_2_, β_3_) is a vector of regression coefficients associated with the vector of the predictors (‘Education/Training’, ‘Health and care’ and ‘Income and purchasing power’), and ε represents the error term.

##### Geographically weighted Poisson regression (GWPR)

Secondly, we developed a GWPR model to investigate the association between hysterectomies and a set of explanatory variables (‘Education/Training’, ‘Health and care’ and ‘Income and purchasing power’), whose parameters can vary from one municipality to another, to predict the incidence of hysterectomy cases. The specification of this model was as follows:

5 $$\ln \left( {\text{Y}} \right) = \ln \left( {E_{i} } \right) + \upbeta_{0} \left( {{\text{u}}_{{\text{i}}} } \right) + \upbeta_{1} \left( {{\text{u}}_{{\text{i}}} } \right)\left( {{\text{ET}}} \right) + \upbeta_{2} \left( {{\text{u}}_{{\text{i}}} } \right)\left( {{\text{IPP}}} \right) + \upbeta_{3} \left( {{\text{u}}_{{\text{i}}} } \right)\left( {{\text{HC}}} \right) + \upvarepsilon$$

Of note, $\beta_{1} ,\beta_{2} , \beta_{3}$ are functions of the location $u_{i} = \left( {u_{xi} ,u_{yi} } \right)$ designating the two-dimensional coordinates of the *i*th municipality. This means that the parameter $\beta = \left( {\beta_{1} ,\beta_{2} , \beta_{3} } \right)$, estimated in Eq. (5), may differ between municipalities. Thus, in the GWPR modeling strategy, the spatial heterogeneity is taken into account, and the parameter β can be expressed in matrix form.

6 $$\left[ {\begin{array}{*{20}c} {\upbeta_{0} \left( {{\text{u}}_{{{\text{x}}1}} ,{\text{u}}_{{{\text{y}}1}} } \right)} & {\upbeta_{1} \left( {{\text{u}}_{{{\text{x}}1}} ,{\text{u}}_{{{\text{y}}1}} } \right)} & {\upbeta_{2} \left( {{\text{u}}_{{{\text{x}}1}} ,{\text{u}}_{{{\text{y}}1}} } \right)} & {\upbeta_{3} \left( {{\text{u}}_{{{\text{x}}1}} ,{\text{u}}_{{{\text{y}}1}} } \right)} \\ {\upbeta_{0} \left( {{\text{u}}_{{{\text{x}}2}} ,{\text{u}}_{{{\text{y}}2}} } \right)} & {\upbeta_{1} \left( {{\text{u}}_{{{\text{x}}2}} ,{\text{u}}_{{{\text{y}}2}} } \right)} & {\upbeta_{2} \left( {{\text{u}}_{{{\text{x}}2}} ,{\text{u}}_{{{\text{y}}2}} } \right)} & {\upbeta_{3} \left( {{\text{u}}_{{{\text{x}}2}} ,{\text{u}}_{{{\text{y}}2}} } \right)} \\ \cdots & \cdots & \cdots & \cdots \\ {\upbeta_{0} \left( {{\text{u}}_{{{\text{xn}}}} ,{\text{u}}_{{{\text{yn}}}} } \right)} & {\upbeta_{1} \left( {{\text{u}}_{{{\text{xn}}}} ,{\text{u}}_{{{\text{yn}}}} } \right)} & {\upbeta_{2} \left( {{\text{u}}_{{{\text{xn}}}} ,{\text{u}}_{{{\text{yn}}}} } \right)} & {\upbeta_{3} \left( {{\text{u}}_{{{\text{xn}}}} ,{\text{u}}_{{{\text{yn}}}} } \right)} \\ \end{array} } \right]$$

In this matrix, $n$ represents the number of municipalities (here, 262). The parameters for each municipality, which form a line in the matrix of Eq. (6), were estimated as by Fotheringham et al. [31].

7 $$\hat{\beta }\left( {\text{i}} \right) = \left( {{\mathbf{X}}^{{\text{T}}} {\mathbf{W}}\left( {{\text{u}}_{{{\text{xi}}}} ,{\text{u}}_{{{\text{yi}}}} } \right){\mathbf{X}}} \right)^{ - 1} {\mathbf{X}}^{{\text{T}}} {\mathbf{W}}\left( {{\text{u}}_{{{\text{xi}}}} ,{\text{u}}_{{{\text{yi}}}} } \right){\mathbf{Y}}$$

In Eq. (7), ${\mathbf{W}}\left( {{\text{u}}_{{{\text{xi}}}} { },{\text{u}}_{{{\text{yi}}}} } \right){ }$ denotes a spatial weight matrix $n$ by $n$, which can be expressed by $W \left( i \right):$

$${\text{W}}\left( {\text{i}} \right) = \left[ {\begin{array}{*{20}c} {{\text{w}}_{{{\text{i}}1}} } & 0 & 0 \\ 0 & {{\text{w}}_{{{\text{i}}2}} } & 0 \\ { \ldots ..} & { \ldots \ldots \ldots \ldots } & { \ldots \ldots } \\ 0 & 0 & {{\text{w}}_{{{\text{in}}}} } \\ \end{array} } \right]$$

where $w_{ij} \left( {j = 1, 2, \ldots , 262} \right)$ is the weight given to the municipality *j* in the model adjustment for municipality *i*.

In this model, our regression equation was estimated for each municipality based on observations in neighboring municipalities. The estimation process was repeated for all the municipalities, and each municipality was weighted by its distance from the regression point. As a result, data from municipalities closer to the municipality where the regression was implemented had more significant weight than data from remote municipalities. In other words, observations closer to municipality $i$ had more influence on the parameter estimation for $\beta_{1} \left( {u_{i} } \right),\beta_{2} \left( {u_{i} } \right),\beta_{3} \left( {u_{i} } \right)$ than those remote from $i$. This influence around $i$ is described by the weighting function $w_{ij}$. In this search, the bi-square kernel below was used for the calculation of $w_{ij}$.

8 $${\text{Bi - square}}:{\text{ w}}_{{{\text{ij}}}} = \left\{ {\begin{array}{*{20}l} {\left| {1 - \left( {\frac{{\left\| {\begin{array}{*{20}c} {{\text{u}}_{{\text{i}}} } & {{\text{u}}_{{\text{j}}} } \\ \end{array} } \right\|}}{{{\text{G}}_{{\text{i}}} }}} \right)^{2} } \right|^{2} } \hfill & {\quad if\;\left\| { \begin{array}{*{20}c} {{\text{u}}_{{\text{i}}} } & {{\text{u}}_{{\text{j}}} } \\ \end{array} } \right\| < {\text{G}}_{{\text{i}}} } \hfill \\ 0 \hfill & {\quad otherwise} \hfill \\ \end{array} } \right.$$

The parameter $Gi$ is a quantity known as the bandwidth. When $G_{i}$ tends towards infinity, $w_{ij}$ approaches 1 and the GWPR becomes a global model, as expressed in Eq. (8). The bi-square kernel was used for the calculation of $w_{ij}$ to avoid high standard errors and unpredictable results in the parameter estimation for municipalities with very few data. This bi-square kernel allows the weighting variable to vary spatially according to the density of the data. Thus, bandwidths are greater where data are scarce and smaller where data are abundant. During the modeling procedure, all of the data of our study were used to determine the bandwidth, to take into account the fact that some municipalities had small populations.

##### Testing spatial autocorrelations of model residuals

Spatial autocorrelation occurs when data from one municipality correlate with data from other neighboring municipalities. Moran's index I was used in this research to evaluate the spatial autocorrelation of the residuals of the model [32]. A negative (positive) value of Moran's I indicates a negative (positive) spatial autocorrelation between municipalities. Moran's I’s values range from − 1 (indicating perfect dispersion) to + 1 (perfect correlation); 0 indicates a random spatial pattern. The Moran's I was considered significant if the associated *p* value was less than 0.05. We also took the spatial autocorrelation into account in all our models.

All the analyses were performed using the R statistical software, and the “SPGWR” package was used to fit the GWPR model.

## Results

### Characteristics of the study population

Table 2 summarizes the characteristics of the women aged 15+ living in Wallonia who underwent hysterectomies between 2012 and 2014. A total of 6905 hysterectomies were performed in Wallonia during the 3-year period. The distribution of the sample by year did not show any significant difference (34.2%, 32.7% and 33.1%, respectively). The vaginal type of procedure was chosen for 56.7% of the women, and the abdominal type, for 43.3%. The main indications for hysterectomy were leiomyomas and benign neoplasms of the female genital tract (55.9%), followed by endometriosis (28.1%) and genital prolapse (16.0%). The highest hysterectomy rate was in the 45–54-year-old age group (46.6%), followed by the 35–44-year-old group (26.2%).

**Table 2:** Number of hysterectomies (N = 6905) and percentage of hysterectomies by age group, year, diagnosis and procedure

	No. of hysterectomies	Percentage
Age-groups		
< 35 years	102	1.5
35–44 years	1807	26.2
45–54 years	3221	46.6
55–64 years	854	12.4
≥ 65 years	921	13.3
Years		
2012	2364	34.2
2013	2257	32.7
2014	2284	33.1
Diagnosis		
Leiomyomas and benign neoplasms of the female genital tract	3859	55.9
Endometriosis	1943	28.1
Genital prolapses	1103	16.0
Type of procedure		
Vaginal hysterectomies	3914	56.7
Abdominal hysterectomies	2991	43.3

### Hysterectomy standardized incidence ratio (SIR) estimation

**Fig. 2:**
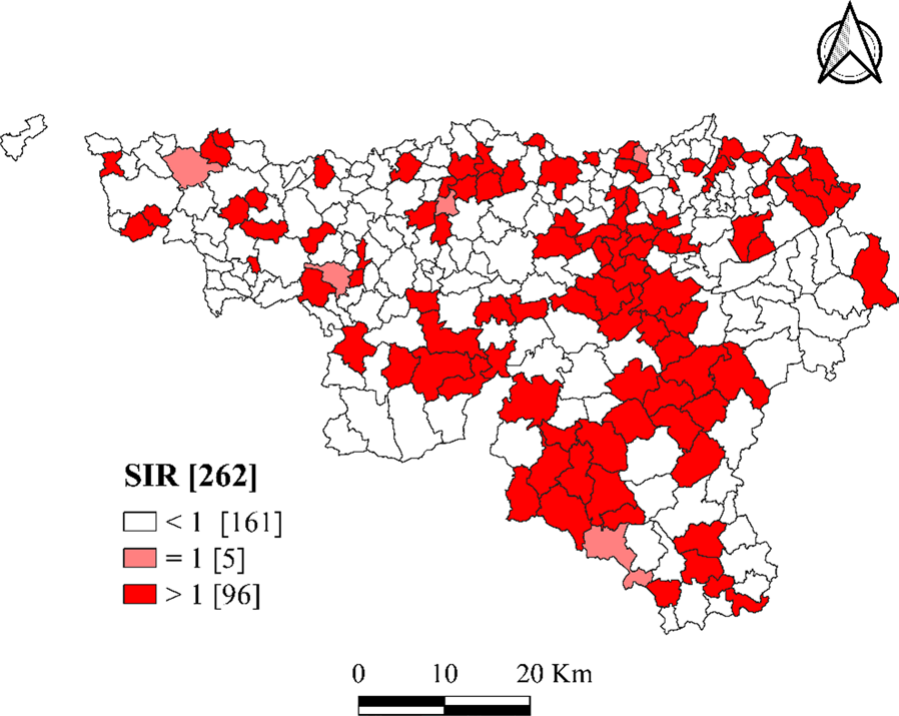
Distribution of the hysterectomy standardized incidence ratio (SIR) in Wallonia, 2012–2014 ( Source: authors)

The overall hysterectomy SIR was 1.101 (95%CI 1.002–1.199) and differed between municipalities in Wallonia. Indeed, it was greater than 1 in 96 municipalities, indicating that the number of cases observed was higher than expected in these municipalities. It was lower than expected in 161 municipalities in Wallonia (Fig. 2). All the provinces showed low values with clusters of higher values. A sizable cluster of 38 municipalities with values higher than 1 was seen on the Eastern part of the map running from North to South across three provinces (Liège, Namur and Luxembourg).

### Multivariate analysis

#### Summary of parameters in the Poisson regression model

Table 3 displays the results for the Poisson regression model. The variables ‘Income and purchasing power’ and ‘Health and care’ were significantly associated with the occurrence of hysterectomy in women aged 15+ in Wallonia. The association with ‘Income and purchasing power’ was positive; if the ‘Income and purchasing power’ score increased, the probability of hysterectomy increased. The association with ‘Health and care’ was negative; meaning that if the ‘Health and care’ score decreased, the probability of hysterectomy increased. The variable ‘Education/Training’ was not significantly associated with the occurrence of the surgery.

**Table 3:** Summary of parameters in the Poisson regression model

Variables	Coefficient	Pr (>|z|)
Education/training	− 0.12	0.17
Income and purchasing power*	0.59	*P* < 0.001
Health and care*	− 0.68	*P* < 0.001

*Results statistically significant

#### Summary of local parameters in the GWPR

**Fig. 3:**
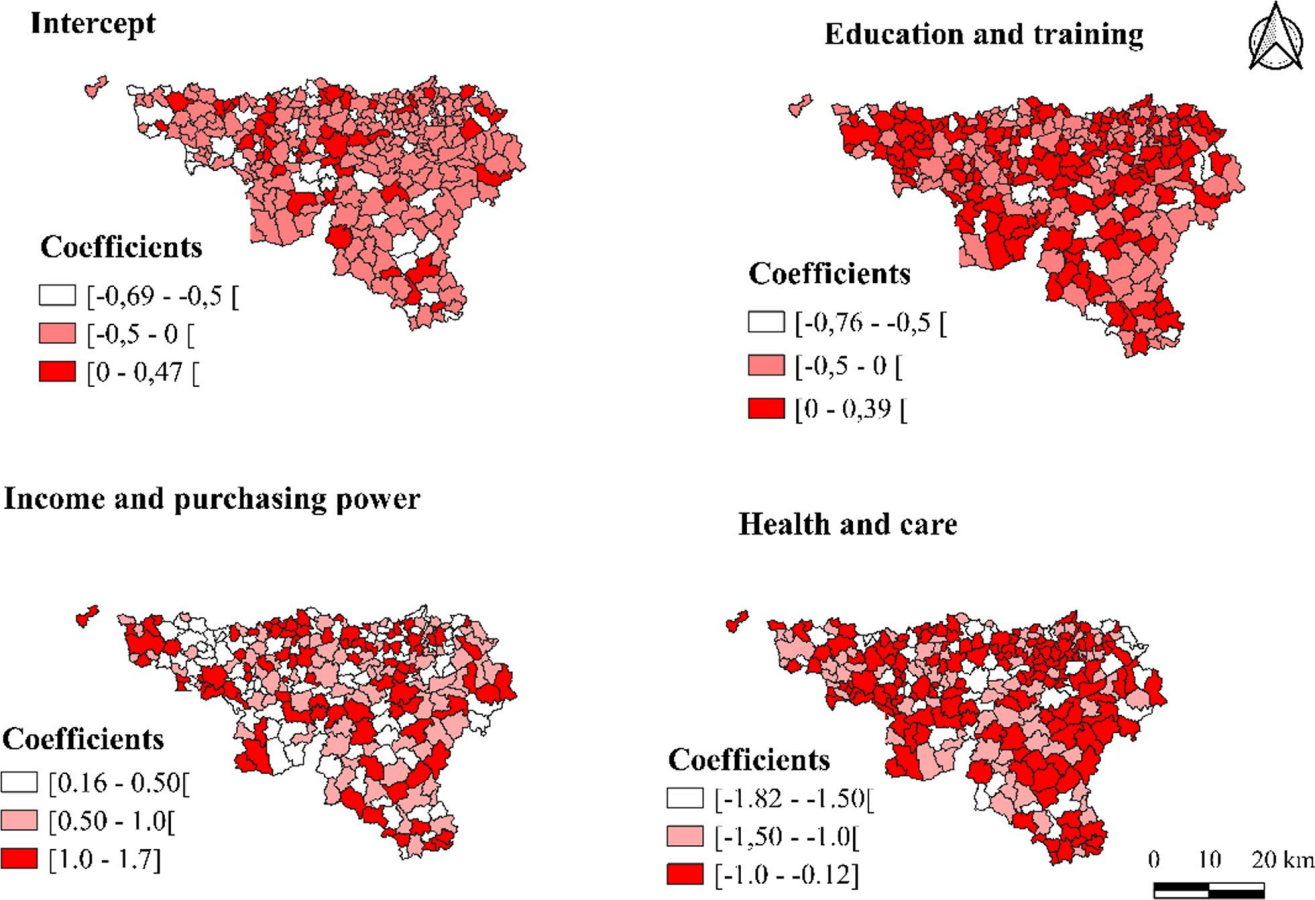
Parameters of predicting variables by municipality in the GWPR ( Source: authors)

Table 4 presents a summary of the parameter estimates in the GWPR model. The local parameters are described by the five indicators statistics (the minimum, first quartile, median, third quartile and maximum values). The distributions of the parameters of the predictive variables over the 262 municipalities of Wallonia are shown in Fig. 3; the red color on the map indicates a higher value of a local parameter estimate. The parameters show clear patterns of spatial variation. The maps indicate that the four parameter estimates are not equal for all locations. The local parameter estimates of the intercept were the highest in the following municipalities: Aiseau-Presles, Andenne, Anderlues, Ans, Antoing, Braine-l'Alleud, Courcelles, Enghien, Eupen, Fontaine-l'Eveque, Frasnes-lez-Avaing, Gedinne, Geer, Grâce-Hollogne, Ham-sur-Heure-Nalinnes, Hastière, Herbeumont, Huy, Incourt, Jalhay, Jodoigne, La Bruyère, La Louvière, Léglise, Marche-en-Famenne, Namur, Nivelles, Orp-Jauche, Oupeye, Philippeville, Plombières, Profondeville, Saint-Léger, Sankt-Vith, Sambreville, Seneffe, Silly, Tintigny and Yvoir. This indicates that women in those municipalities are more likely to undergo hysterectomies when the other variables are kept constant. However, the local parameters were lower in most of the municipalities: Anhee, Aubange, Baelen, Braine-le-Château, Braine-le-Comte, Brunehaut, Chatelet, Chaudfontaine, Chaumont-Gistoux, Ciney, Estinnes, Etalle, Farciennes, Fexhe-le-Haut-Clocher, Floreffe, Florennes, Frameries, Grez-Doiceau, Hensies, Honnelles, Kelmis, La Hulpe, La Roche-en-Ardenne, Libramont-Chevigny, Meix-devant-Virton, Mettet, Mons, Mont-de-l'Enclus, Morlanwelz, Mouscron, Nandrin, Neufchateau, Onhaye, Ouffet, Quiévrain, Rixensart, Sombreffe, Soumagne, Tellin, Tournai, Vaux-sur-Sûre, Villers-la-Ville, Villers-le-Bouillet and Wasseige. This indicates a weak relationship with undergoing hysterectomy (Fig. 3). For the ‘Education/training’, ‘Health and care’ and ‘Income and purchasing power’ scores, the parameters changed from one municipality to another. The association between hysterectomy and ‘Education/training’ showed the largest spatial variation across the region, as indicated by the coefficient range (− 0.764 to 0.39). ‘Education/training’ was negatively associated with the occurrence of hysterectomy in women in 136 municipalities in Wallonia, while it was positively associated in the other 126 municipalities. In addition, the local parameter estimates of ‘Education/training’ were the lowest in the following municipalities: Anhee, Berloz, Celles, Colfontaine, Ecaussines, Esneux, Florenville, Froidchapelle, Gembloux, Hamois, Heron, Hotton, Libin, Limbourg, Malmedy, Messancy, Mettet, Modave, Rebecq, Rochefort, Vielsalm, Waimes, and Wavre; they were the highest in the following municipalities: Anthisnes and Wanze. Higher local parameter estimates indicate that the women living in those municipalities would be more likely to undergo hysterectomies (Fig. 3). The local parameter estimates for ‘Health and care’ were relatively low in all the municipalities. The map shows that all of the local parameter estimates for the ‘Health and care’ variable are negative. This indicates that increasing access to ‘Health and care’ reduces the number of hysterectomies in each municipality. The local parameter estimates for ‘Health and care’ were the lowest in the following municipalities: Anhee, Baelen, Celles, Honnelles, La Hulpe, Limbourg, Messancy and Mons (Fig. 3). The local parameter estimates for ‘Income and purchasing power’ were the highest in Bouillon, Ciney, Ensies, and Musson, and the lowest in Marche-en-Famenne, Orp-Jauche and Profondeville. The map shows that all the local parameter estimates for the ‘Income and purchasing power’ variable are positive; this indicates that increasing the ‘Income and purchasing power’ of women increases the number of hysterectomies for each municipality (Fig. 3).

**Table 4:** Summary of local parameters in the GWPR

Variables	Minimum	Lower quartile	Median	Upper quartile	Maximum
Intercept	− 0.685	− 0.44	− 0.242	− 0.07	0.468
Education/training	− 0.764	− 0.308	− 0.032	0.182	0.39
Income and purchasing power	0.167	0.444	0.633	1.068	1.69
Health and care	− 1.802	− 1.308	− 0.856	− 0.284	− 0.12

## Discussion

This study described the geographical distribution of hysterectomy incidence in women aged 15+ in Wallonia and demonstrated the potential of GWPR to improve our understanding of the factors that influence the use of this surgery. The hysterectomy incidence varied across Wallonia. Although the Poisson regression model performed well, it ignored the spatial context, as it represented only the overall relation for the whole of Wallonia, whereas GWPR was able to represent local variation in the relationships between hysterectomy incidence and socioeconomic factors that play a role in each municipality. The results from the GWPR allow a deeper understanding of the spatial variations, as hysterectomy incidence was shown to have a nonstationary relationship with socioeconomic factors. This will help in building tailored recommendations to diminish the disparities.

The mapping of the SIR (Fig. 2) identified clusters of municipalities with higher and lower hysterectomy incidences compared to the mean in Wallonia, while the maps in Fig. 3 show a different spatial distribution of hysterectomies according to predicting variables in the GWPR. This means that taking into account one by one the factors influencing the use of the surgery is necessary for implementing preventive measures to reduce surgery numbers and target at-risk populations.

Many studies have suggested that hysterectomies tend to be more frequent in the most deprived areas. First, our results demonstrate that women aged 15+ living in a municipality with lower ‘Health and care’ scores were more likely to undergo hysterectomies than those living in a municipality with a higher score. The positive and significant impact of ‘Health and care’ on municipal hysterectomy incidence is consistent with most literature findings [9, 17, 22]. The score used for the variable included, among other factors, accesses to hospital or medical centre. Belgium has an equal distribution of hospital facilities across the country, and people do not face any financial barriers to hospitalization, since the majority of the population is covered by health insurance, and co-payment for hospitalization is very uncommon [33]. However, women with lower socioeconomic statuses living in areas with less access to care are less likely to receive regular gynecological examinations than those with higher socioeconomic statuses, who can choose between several medical offers (public or private). Therefore, it would be interesting to analyze this correlation by age group to investigate if hysterectomy is also seen as a long-term contraceptive method in women who have already achieved their desired family sizes and would like to limit medical control visits and out-of-pocket costs [22]. On the contrary, some studies have demonstrated that women with private healthcare insurance or with more visits to the general practitioner are also more likely to have hysterectomies [17, 23]. This proxy also included several components that were not useful for our analysis (such as ‘Prevalence of diabetes’ or ‘Pedestrian access to a pharmacy’), which could have biased the results.

The second association with ‘Income and purchasing power’ was not as expected. Women aged 15+ living in municipalities with lower ‘Income and purchasing power’ scores were less likely to receive hysterectomies. This association contradicts studies conducted in Belgium in the past decades [1, 9] and other countries indicating that women with lower socioeconomic statuses are more likely to undergo hysterectomies [16, 21, 22]. By contrast, in Ontario, a positive association was demonstrated for those on middle-quintile incomes [19]. The authors suggested that the decision for surgery is complex and there might be some reluctance from the middle-quintile incomes to pay for more costly conservative treatments and challenge for the lower-quintiles in accessing surgical care. Three studies in Finland found a positive association between income and hysterectomy rates [2, 34, 35]. This association was also observed in other studies conducted in the United States [23, 36] and in Australia, with more surgeries in women with private health insurances [17]. Some of the explanations put forward were an overuse of surgeries in women with a complementary private health care insurances or in well-off women who can afford private practices costs not reimbursed by social security, in light of possible financial incentive for the physicians in non-public hospital [17, 23]. Some studies also analyses the relation in regards to the pathology treated, and this relation was different between the different socio-economic groups indicating a better access to gynecological center for detection and diagnosis for some hysterectomies indications [2, 34]. The reasons behind this association deserve more exploration, as it may reflect complexities in the underlying reasons behind decisions to proceed with surgery, such as cultural beliefs or fears of the potential additional costs of several medical visits in deprived women [33].

For ‘Education/training’, the fact that the estimated coefficients ranged from negative to positive over the study area did not emerge with traditional regression, showing the importance of the GWPR specification [37]. The association between education and hysterectomy is not always negative. This spatial heterogeneity in the municipal associations indicates how the factor might have a greater effect on hysterectomy incidence in some municipalities than others. Several studies have demonstrated that women with lower education levels are more likely to undergo hysterectomies [3, 38, 39]. Such women have less knowledge of the therapeutic options available for their conditions, assuming that the physician knows best, and are less confident in discussing alternatives with their healthcare professionals [21]. Some healthcare professionals have difficulty discussing sexual and reproductive health with less educated women. These situations can lead physicians to make decisions on behalf of their patients, without involving them [21, 40, 41]. This dimension included 2 variables that are proxies for the Education levels (‘Percentage of pupils on-time in secondary education’ and ‘Access to a nursery or primary school’) that could bias the results.

Our findings should be interpreted in the context of several limitations. First, the ICWB score is inspired by the Canadian Index of Well-being and covers all aspects of wellbeing, not being limited to health or socioeconomic correlates (i.e., it includes dimensions such as municipal environment and communication); as such, this indicator is unique to Wallonia. Indeed, an equivalent indicator is not available in two other Belgian regions, which limits comparison at a national level. The ICWB index does not give a breakdown of the correlates by age group or by sex, which also limits comparison with other studies. However, there are advantages of using ICWB correlates, as they are issued from the same index and include a large selection of dimensions available for the same geographical entity. This limits the sources for data collection, uses the same validated method, and allows the same process of calibration for the dimensions.

Another identified limitation was the use of the aggregate data to estimate the hysterectomy rate. Indeed, the denominator used for hysterectomy rates, i.e., the number of women aged 15+ in a municipality, included women who had already undergone hysterectomies prior to the period of our study or for indications other than those selected in the frame of this study (e.g., cancer and postpartum hemorrhage). These women were no longer at risk of having the surgery. This could have led to an underestimation of the SIR [22, 42].

The last limitation was the design of the ecological studies, limiting the ability to prove causality. Further analysis is required to explore the underlying causes of these variations. Alternative variables could also be applied to explore their impact on hysterectomy incidence, such as exploring the degree of satisfaction of the patients and complications after a hysterectomy to assess the benefits and risks of radical surgeries. Further studies to analyze whether the contemporary decrease in crude hysterectomy rates in Belgium, as exposed in the recent National Institute for Health and Disability Insurance report [8], is correlated with the use of alternative treatments would be useful. Hysterectomy is a common surgery and provides relief for a number of benign gynecological problems, but it is often associated with negative psychological, sexual and physical outcomes [5, 43]. Various alternatives to hysterectomy are available and should be preferred to the radical surgery. Hysterectomy is recommended when other treatment options have failed, are contraindicated, or are declined by women [43, 44]. The influence of the socioeconomic level on the incidence of hysterectomy could be due to several factors. Women with low levels of education may lack knowledge of less invasive methods, consult a healthcare professional later, or have variable access to healthcare [45]. Some studies have demonstrated that the sex, age and academic practices of the gynecologist play a role in the indication for hysterectomy [23, 46]. A public information campaign in the mass media conducted in Switzerland in 1984 led to a drop in hysterectomy rates after the start of the campaign and during the following year [43]. Today, websites created by lay women associations and patient support groups on social media are available and provide simple explanations of alternatives to hysterectomy, also providing advice to empower women regarding their health. These forms of support can be used to facilitate discussion with gynecologists or healthcare professionals before deciding if radical surgery is the best therapeutic option [21, 47]. Strategies that should be adopted include clinical education, the generation of good practice guidelines, peer review, feedback to physicians on clinical practice profiles, public education, and improving the understanding of patient preferences in the decision process [11, 21, 33, 44]. Physicians and gynecologists should adopt a holistic approach with space for discussion on the psychological impact of uterus resection on a woman’s wellbeing. Outcomes that matter to patients should not be ignored in the discussion, such as the relief of symptoms, long-term complications (with/without surgery) and improvement of quality of life [11]. A focused approach, encompassing the understanding of the local socioeconomic correlates, would optimize the decision-making processes of women and their caregivers and prevent the overuse of the procedure for at-risk women.

## Conclusions

Low interregional variation is generally presumed to indicate optimal medical services [14]. It was demonstrated that the rates of hysterectomy varied spatially across Wallonia, in Belgium. Moreover, hysterectomy incidence was found to be associated with a high score for ‘Income and purchasing power’, a low score for ‘Health and care’, and various scores regarding ‘Education/training’. Spatial variation in the relationships between hysterectomy incidence and socioeconomic variables means that in some municipalities, those socioeconomic variables have greater effects on the incidence than in the other places. Ignoring those spatial variations could lead to inefficient resource usage. Our study identified persistent differences in the use of hysterectomies between the municipalities in Wallonia, which might represent inefficiency or inequality in the provision of care. There is concern about the potential abuse of hysterectomy for benign diseases, considering the possible short-term complications and long-term deleterious effects. Despite the fact that Belgium has a good healthcare system, social inequalities in health remain in the population [33]. It is important to quantify inequalities in the use of hysterectomies and their causes in order to take steps to address them at the local level of care policy. In the current study, the use of GWPR was proposed to increase the consideration of the local nature of ecological associations and enable a deeper understanding of the situation at the municipal level. These findings would help health policy to move beyond providing uniform recommendations for all municipalities, as regional disparities are large. Considering the fact that municipal-target intervention is difficult to conduct practically, a local understanding of the socioeconomic factors influencing the use of the surgery would help healthcare professionals to act efficiently and target information campaigns. Our results offer further evidence for enhancing programs based on geographical variations and assisting women at risk to make the best choices.

## Data Availability

The datasets extracted from RHM analysed during the current study are not publicly available but are available from the corresponding author on reasonable request and with permission of AViQ.

## Abbreviations

15+: Older than 15-year-old
AViQ: Walloon agency for a life of quality
CI: Confidence interval
GLM: Generalized linear model
GWPR: Geographically weighted Poisson regression
ICD: International classification of diseases
ICWB: Index of conditions of well-being
IWEPS: Walloon institute of evaluation, prospective and statistics
RHM: Minimum hospital summary
SIR: Standardized incidence ratio

## References

[1] Diels J, Cluyse L, Gaussin C, Mertens R. L’hystérectomie en Belgique. Mutualités Chrétiennes; 1999. Report No.: 1.

[2] Luoto R, Keskimaki I, Reunanen A (1997). Socioeconomic variations in hysterectomy: evidence from a linkage study of the Finnish hospital discharge register and population census. J Epidemiol Community Health.

[3] Wilson LF, Mishra GD (2016). Age at menarche, level of education, parity and the risk of hysterectomy: a systematic review and meta-analyses of population-based observational studies. PLoS ONE.

[4] Cosson M, Dubecq F, Debodinance P, Querleu D, Crépin G. Hystérectomies: indications, voies, conservations annexielles ou cervicales. 1996. pp. 253–280. **(Extrait des Mises à jour en Gynécologie et Obstétrique)**.

[5] Carlson KJ, Nichols DH, Schiff I (1993). Indications for hysterectomy. N Engl J Med.

[6] Wilson L, Pandeya N, Byles J, Mishra G (2018). Hysterectomy and incidence of depressive symptoms in midlife women: the Australian longitudinal study on women’s health. Epidemiol Psychiatr Sci.

[7] Hanstede MMF, Burger MJ, Timmermans A, Burger MPM (2012). Regional and temporal variation in hysterectomy rates and surgical routes for benign diseases in the Netherlands. Acta Obstet Gynecol Scand.

[8] Meeus P, Dalcq V, Van Geystelen A. Variations de pratiques médicales—Hystérectomie. INAMI; 2019 mars. https://www.belgiqueenbonnesante.be/images/INAMI/Rapports/RAPPORT-FR-HYSTERECTOMIE_2015-2017.pdf.

[9] Jacques J, Gillain D, Fecher F, Van De Sande S, Vrijens F, Ramaekers D, et al. Etude des disparités de la chirurgie élective en Belgique. Good Clinical Practice (GCP). Bruxelles: Centre fédéral d'expertise des soins de santé (KCE). 2006. KCE reports 42B (D/2006/10.273/46).

[10] Cromwell DA, Mahmood TA, Templeton A, van der Meulen JH (2009). Surgery for menorrhagia within English regions: variation in rates of endometrial ablation and hysterectomy. BJOG Int J Obstet Gynaecol.

[11] Lefebvre G, Allaire C, Jeffrey J, Vilos G (2018). No 109-Hysterectomy. J Obstet Gynaecol Can.

[12] Wennberg J (1973). Gittelsohn null. Small area variations in health care delivery. Science.

[13] Yusuf F, Leeder S, Wilson A (2016). Recent estimates of the incidence of hysterectomy in New South Wales and trends over the past 30 years. Aust N Z J Obstet Gynaecol.

[14] Lauterbach R, Joseph M, Haklai Z, Gil L, Lowenstein L (2019). Geographic variation of hysterectomy rates in the Israeli health care system during the years 2007–2016. Isr J Health Policy Res.

[15] Cooper R, Lucke J, Lawlor DA, Mishra G, Chang J-H, Ebrahim S (2008). Socioeconomic position and hysterectomy: a cross-cohort comparison of women in Australia and Great Britain. J Epidemiol Community Health.

[16] Erekson EA, Weitzen S, Sung VW, Raker CA, Myers DL (2009). Socioeconomic indicators and hysterectomy status in the United States, 2004. J Reprod Med.

[17] Byles JE, Mishra G, Schofield M (2000). Factors associated with hysterectomy among women in Australia. Health Place.

[18] Cooper R, Lawlor DA, Hardy R, Ebrahim S, Leon DA, Wadsworth MEJ (2005). Socio-economic position across the life course and hysterectomy in three British cohorts: a cross-cohort comparative study. BJOG Int J Obstet Gynaecol.

[19] Chen I, Wise MR, Dunn S, Anderson G, Degani N, Lefebvre G (2017). Social and geographic determinants of hysterectomy in Ontario: a population-based retrospective cross-sectional analysis. J Obstet Gynaecol Can.

[20] Settnes A, Jorgensen T (1996). Hysterectomy in a Danish cohort. Prevalence, incidence and socio-demographic characteristics. Acta Obstet Gynecol Scand.

[21] Materia E, Rossi L, Spadea T, Cacciani L, Baglio G, Cesaroni G (2002). Hysterectomy and socioeconomic position in Rome, Italy. J Epidemiol Community Health.

[22] Gartner DR, Doll KM, Hummer RA, Robinson WR (2018). Contemporary geographic variation and sociodemographic correlates of hysterectomy rates among reproductive-age women. South Med J.

[23] Geller SE, Burns LR, Brailer DJ (1996). The impact of nonclinical factors on practice variations: the case of hysterectomies. Health Serv Res.

[24] Spilsbury K, Semmens JB, Hammond I, Bolck A (2006). Persistent high rates of hysterectomy in Western Australia: a population-based study of 83 000 procedures over 23 years. BJOG Int J Obstet Gynaecol.

[25] Kjerulff K, Langenberg P, Guzinski G (1993). The socioeconomic correlates of hysterectomies in the United States. Am J Public Health.

[26] Nakaya T, Fotheringham AS, Brunsdon C, Charlton M (2005). Geographically weighted Poisson regression for disease association mapping. Stat Med.

[27] Handbook of Spatial Analysis | Insee. https://www.insee.fr/en/information/3635545.

[28] be.STAT. https://bestat.statbel.fgov.be/bestat/crosstable.xhtml?view=c1649c18-ea66-4286-9310-2413e74134f8.

[29] Indicateurs complémentaires au PIB: L’indice des conditions de bien-être (ICBE). Iweps. https://www.iweps.be/publication/indicateurs-complementaires-pib-lindice-conditions-de-bien-etre-icbe/.

[30] Cheng EMY, Atkinson PM, Shahani AK (2011). Elucidating the spatially varying relation between cervical cancer and socio-economic conditions in England. Int J Health Geogr.

[31] Fotheringham AS, Brunsdon C, Charlton M (2003). Geographically weighted regression: the analysis of spatially varying relationships.

[32] Tango T (2010). Statistical methods for disease clustering.

[33] Meeus P, Haelterman M. Belgium: geographic variations in health care. In: Geographic variations in health care. OECD; 2014. p. 113–36. https://www.oecd-ilibrary.org/social-issues-migration-health/geographic-variations-in-health-care/belgium-geographic-variations-in-health-care_9789264216594-6-en.

[34] Keskimäki I, Salinto M, Aro S (1996). Private medicine and socioeconomic differences in the rates of common surgical procedures in Finland. Health Policy Amst Neth.

[35] Manderbacka K, Arffman M, Leyland A, McCallum A, Keskimäki I (2010). Change and persistence in healthcare inequities: access to elective surgery in Finland in 1992–2003. Scand J Public Health.

[36] Carlisle DM, Valdez RB, Shapiro MF, Brook RH (1995). Geographic variation in rates of selected surgical procedures within Los Angeles County. Health Serv Res.

[37] Alves ATJ, Nobre FF, Waller LA (2016). Exploring spatial patterns in the associations between local AIDS incidence and socioeconomic and demographic variables in the state of Rio de Janeiro, Brazil. Spat Spatio-Temporal Epidemiol.

[38] Dharmalingam A, Pool I, Dickson J (2000). Biosocial determinants of hysterectomy in New Zealand. Am J Public Health.

[39] Marks NF, Shinberg DS (1997). Socioeconomic differences in hysterectomy: the Wisconsin longitudinal study. Am J Public Health.

[40] Coulter A (1999). Paternalism or partnership? Patients have grown-up and there's no going back. BMJ..

[41] Médecins du monde, INAMI. Livre Vert sur l’accès aux soins en Belgique. Wolters Kluwer Belgium SA; 2014. p. 362. https://www.inami.fgov.be/SiteCollectionDocuments/livre-vert.pdf.

[42] Stang A, Kluttig A, Moebus S, Völzke H, Berger K, Greiser KH (2014). Educational level, prevalence of hysterectomy, and age at amenorrhoea: a cross-sectional analysis of 9536 women from six population-based cohort studies in Germany. BMC Womens Health.

[43] Domenighetti G, Luraschi P, Casabianca A, Gutzwiller F, Spinelli A, Pedrinis E (1988). Effect of information campaign by the mass media on hysterectomy rates. Lancet.

[44] Lefebvre G (2003). Hystérectomie: le droit de choisir. J Obstet Gynaecol Can.

[45] Prütz F, Knopf H, von der Lippe E, Scheidt-Nave C, Starker A, Fuchs J (2013). Prevalence of hysterectomy in women 18 to 79 years old: results of the German health interview and examination survey for adults (DEGS1). Bundesgesundheitsblatt Gesundh Gesundheit.

[46] Bickell NA, Earp JA, Garrett JM, Evans AT (1994). Gynecologists’ sex, clinical beliefs, and hysterectomy rates. Am J Public Health.

[47] Wade J, Pletsch PK, Morgan SW, Menting SA (2000). Hysterectomy: What do women need and want to know?. J Obstet Gynecol Neonatal Nurs.

